# Prediction of pre-hospital blood transfusion in trauma patients based on scoring systems

**DOI:** 10.1186/s12873-022-00770-x

**Published:** 2023-01-12

**Authors:** Michal Plodr, Jana Berková, Radomír Hyšpler, Anatolij Truhlář, Jiří Páral, Jaromír Kočí

**Affiliations:** 1 Faculty of Military Health Sciences, University of Defence, 50001 Hradec Kralove, Czech Republic; 2 Emergency Medical Services of the Hradec Kralove Region, 50012 Hradec Kralove, Czech Republic; 3 Department of Emergency Medicine, Charles University in Prague, University Hospital Hradec Kralove, 50003 Hradec Kralove, Czech Republic; 4 Department of Clinical Chemistry, Charles University in Prague, University Hospital Hradec Kralove, 50003 Hradec Kralove, Czech Republic; 5 Department of Anestesiology and Intensive Care Medicine, Charles University in Prague, University Hospital Hradec Kralove, 50003 Hradec Kralove, Czech Republic; 6 Department of Surgery, Charles University in Prague, University Hospital Hradec Kralove, 50003 Hradec Kralove, Czech Republic

**Keywords:** Pre-hospital, Transfusion, Trauma, Shock index, Pulse pressure, Scoring systems

## Abstract

**Background:**

Pre-hospital blood transfusion (PHBT) is a safe and gradually expanding procedure applied to trauma patients. A proper decision to activate PHBT with the presently limited diagnostic options at the site of an incident poses a challenge for pre-hospital crews. The purpose of this study was to compare the selected scoring systems and to determine whether they can be used as valid tools in identifying patients with PHBT requirements.

**Methods:**

A retrospective single-center study was conducted between June 2018 and December 2020. Overall, 385 patients (aged [median; IQR]: 44; 24–60; 73% males) were included in this study. The values of five selected scoring systems were calculated in all patients. To determine the accuracy of each score for the prediction of PHBT, the Receiver Operating Characteristic (ROC) analysis was used and to measure the association, the odds ratio with 95% confidence intervals was counted (Fig. 1).

**Results:**

Regarding the proper indication of PHBT, shock index (SI) and pulse pressure (PP) revealed the highest value of AUC and sensitivity/specificity ratio (SI: AUC 0.88; 95% CI 0.82–0.93; PP: AUC 0.85 with 95% CI 0.79–0.91).

**Conclusion:**

Shock index and pulse pressure are suitable tools for predicting PHBT in trauma patients.

## Background

Trauma is the most frequent cause of death in patients under the age of 45 years, and haemorrhage is the most frequent preventable cause of death [1]. Improved outcomes in patients with timely administered blood products have been confirmed [2, 3]. Controversy has still emerged around the questions of whether timely initiated administration of blood products should be launched just in the pre-hospital phase – pre-hospital blood transfusion (PHBT), and whether this procedure is effective due to the initial limitations (logistical support, storage conditions and equipment, limited precise diagnostic options at the site of the incident with the risk of unnecessarily administered blood products). Although intuitively it can be assumed that PHBT should improve survival, up-to-date published data on this topic has shown ambiguous results. Observational studies present promising results in terms of improved patient outcomes, including decreased blood product use, reduced early in-hospital mortality, more efficient expansion of intravascular volume, and reduced risk of early trauma-induced coagulopathy [4–6]. Two randomised trials in 2018 comparing the effect of prehospital blood product administration and standard fluid resuscitation (0.9% chloride saline) revealed contradictory results. Moore et al. did not demonstrate that prehospital plasma use was associated with a survival benefit; on the other hand, Sperry et al. demonstrated that in injured patients at risk of haemorrhagic shock that the prehospital administration of thawed plasma was safe and resulted in lower 30-day mortality and a lower median prothrombin-time ratio than standard care resuscitation [7, 8]. The latest randomized RePHILL trial compared prehospital use of PRBC and lyophilised plasma (LyoPlas) group to 0.9% chloride saline group. The trial did not show, that prehospital PRBC-LyoPlas was superior to 0.9% sodium chloride for adult patients with trauma-related haemorrhagic shock. However, the authors recommend further research in this area to identify the characteristics of patients who might benefit from prehospital transfusion [9]. Therefore, we assume that PHBT is a feasible and safe concept, and that maximum attention should be focused on identifying patients with a need for PHBT as accurately as possible. Although, with the limited tools and capabilities that are available to pre-hospital teams, it has proven to be exceptionally challenging to identify such patients. Isolated vital signs, such as heart rate or systolic blood pressure, have been found to be unreliable in the assessment of hypovolemic shock [10, 11]. During the in-hospital phase, a predictive value of scoring systems in association with internal source activation, mortality risk, and massive transfusion activation has been proved. Presently, around 20 scoring systems have to date existed, but many of them are based on cumbersome calculations that take precious time and involve results from laboratory tests and imaging [12–14].

The objective of this analysis was to evaluate whether the selected scoring systems calculated from variables easily detectable at the incident site (age, mechanism of injury, value of basic vital signs) can be used to identify patients who are suitable for PHBT administration before the initiation of massive transfusion (MT) after admission to the emergency department (ED): Shock index – SI; Age multiplied by shock index – AGE-SI; Pulse pressure – PP; Reverse shock index multiplied by GCS – rSI-G; Mechanism of injury, GCS, age, systolic blood pressure – MGAP.

## Methods

### Study design and setting

**Fig. 1:**
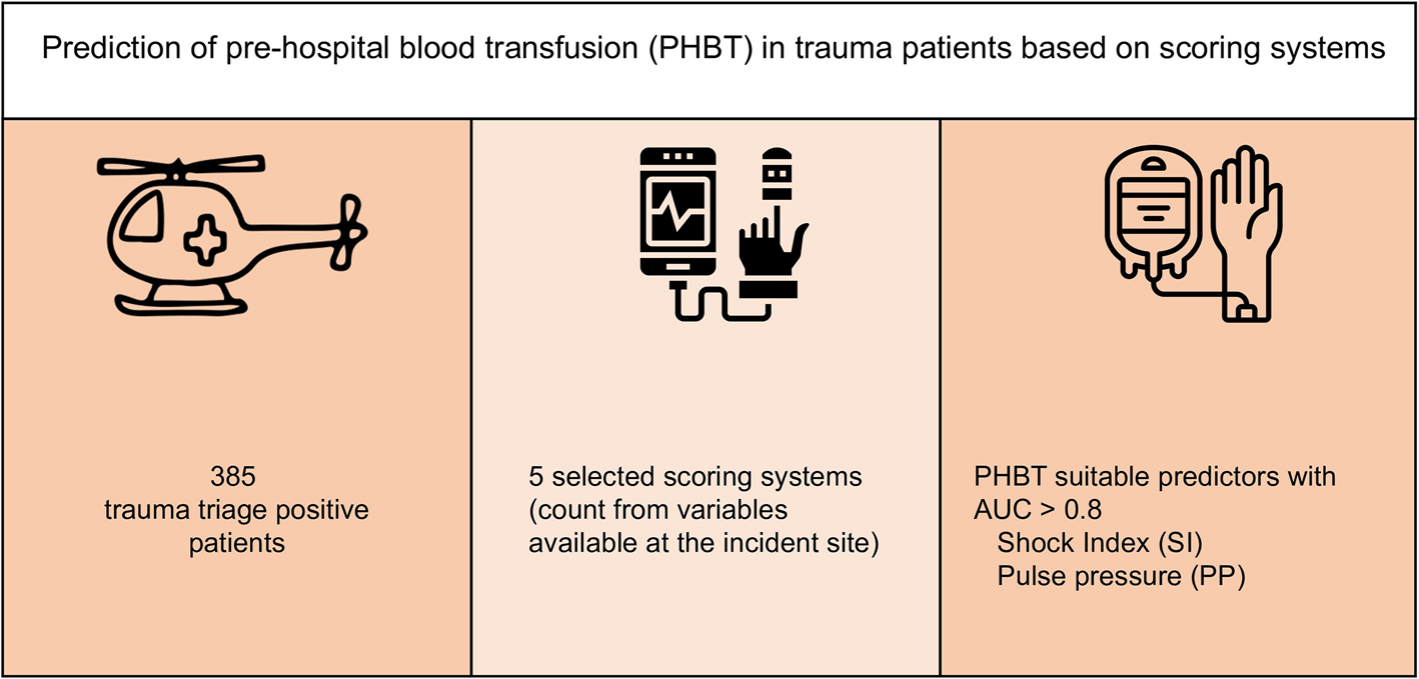
Visual abstract

The retrospective observational single-center analysis was conducted at the Helicopter Emergency Medical Service (HEMS) of the Hradec Kralove region and the Emergency Department, University Hospital, Hradec Kralove – Trauma Center Level I. The HEMS of the Hradec Kralove region is responsible for approximately 10,000 km^2^ with up to 1.1 million citizens. The University Hospital in Hradec Kralove is a facility with 1,300 beds that serves as a high-level center of health care for more than 1 million patients from the eastern part of The Czech Republic. The HEMS has been using the PHBT (Fig. 1) concept since the 1^st^ of June 2018. Until the 31^st^ of May 2020, one unit of O RhD negative red blood cells and one unit of AB plasma were available, and since the 1^st^ of June 2020, two units of Low Titer Group O Whole Blood (LTOWB) have been available. Transfusion units were blood typified in the transfusion department before being placed in the helicopter. Triage-positive patients, who were treated by the HEMS crew and transferred to the ED of the University Hospital in Hradec Kralove, were included in the analysis. Eligible patients for PHBT were considered injured adults, (> 18 years) with findings of a nonpalpable radial pulse or a systolic blood pressure below 90 mmHg, penetrating torso injury, proximal sub amputation and/or amputation, unstable chest, unstable pelvic ring, or the decision was made based on the clinician´s judgement. The number of patients to whom a transfusion was administered or was not administered in the pre-hospital phase, and the number of patients to whom a massive transfusion was or was not activated after admission to the ED were observed. The correct indication for PHBT was considered to be when the massive transfusion was continued after admission to the ED (≥ 4 PRBCs/1 h or ≥ 10 PRBCs/24 h).The decision on MT activation is made at the ED in accordance with Massive Transfusion in Trauma Guidelines [15]. The selected scoring systems were calculated for all patients based on the values of the identified vital signs at the site of the incident.

### Study population

Monitoring was conducted during the period from the 1^st^ of June 2018 to the 31^st^ of December 2020. Patients who were primarily treated by the HEMS and due to triage-positivity required transportation to the trauma center were included in the study. The criteria for the triage-positivity complied with „ Guidelines for field triage of injured patients – steps one and two “ [16]. Patients under 18 years of age, patients transferred to another hospital, patients resuscitated for traumatic out-of-hospital cardiac arrest and declared dead on scene and patients with data loss (missing values for any of the monitored criteria) were excluded from the study.

### Data collection

**Fig. 2:**
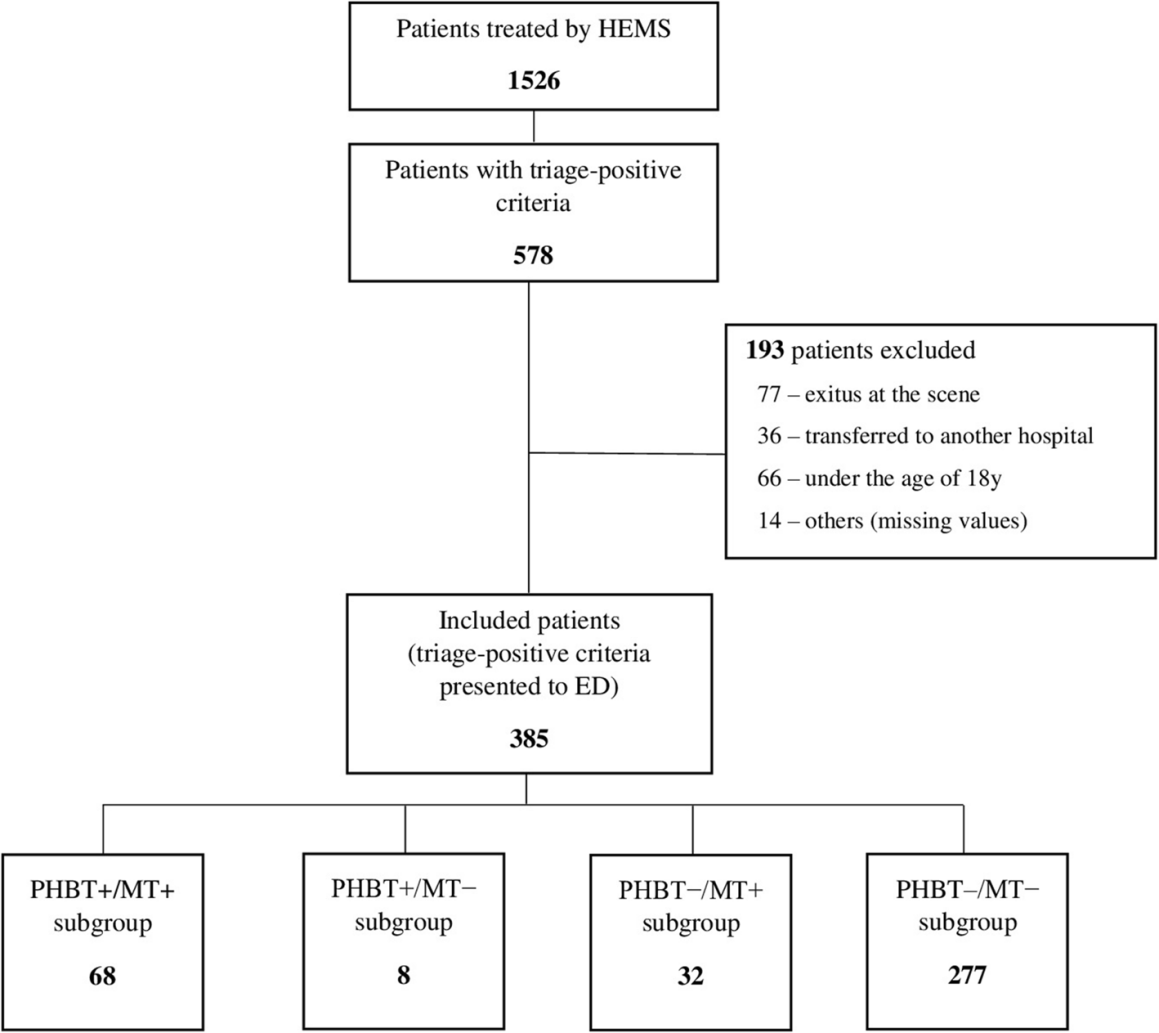
Study flow diagram

The data was extracted from the electronic patient database (EZD^©^, Czech Republic), the platform used by the Emergency Medical Services, and from the hospital information system used at the University Hospital in Hradec Kralove (NIS^©^, Czech Republic). After arrival at the ED, patients were divided into 4 subgroups (Fig. 2) according to the decision on PHBT and subsequent MT activation:

- PHBT + /MT + : PHBT activated, the MT activated after the admission to the ED.
- PHBT + /MT − : PHBT activated, the MT not activated after the admission to the ED.
- PHBT − /MT + : PHBT not activated, the MT activated after the admission to the ED.
- PHBT − /MT − : PHBT not activated, the MT not activated after the admission to the ED.

Based on vital signs values, as well as the age and mechanism of injury, the following scoring systems were calculated in these patients (Table 1).

**Table 1:** Calculation formulas for scoring systems

Scoring system	Variables	Calculation
SI (Shock Index)	Heart rate (HR) Systolic blood pressure (SBP)	HR ÷ SBP
AGE–SI (Age multiplied by Shock Index)	Age Shock index	(HR ÷ SBP) ∙ Age
PP (Pulse Pressure)	Systolic blood pressure Diastolic blood pressure (DBP)	SBP − DBP
rSI–G (reversed Shock Index multiplied by GCS)	Systolic blood pressure Glasgow Coma Scale	(SBP ÷ HR) ∙ GCS
MGAP (Mechanism; GCS; Age; Systolic blood pressure)	Mechanism of injury (M):	M + G + A + P sum of points from each component
	Blunt: + 4 pt Penetrating: 0 pt	
	GCS (G): 3–15 pt AGE (A): < 60y: + 5 pt > 60y: 0 pt	
	Systolic blood pressure (P): > 120 mmHg: + 5 pt 60–120 mmHg: + 3 pt < 60 mmHg: 0 pt	

### Statistical analysis

All statistical analyses were performed using the SigmaStat software version 3.1. (Systat Software Inc., US). The data distribution was tested using the Shapiro–Wilk normality test and the Brown-Forsythe equal variance test. As all the datasets were not normally distributed, the data is presented as the median (interquartile range) and the statistical difference between the groups was tested using a Kruskal–Wallis One Way Analysis of Variance on Ranks and p ≤ 0.05 was considered statistically significant. The receiver operating characteristic (ROC) areas and area comparisons were determined using the DeLong, DeLong and Clarke-Pearson methods.

## Results

Of the 1526 patients treated by HEMS crews, 578 patients met trauma triage-positive criteria at the scene. Of this group, 193 patients were excluded, the highest proportion of those due to exitus at the scene (*n* = 77) and aged less than 18 years (*n* = 66). Finally, we analysed 385 patients, who met the inclusion criteria, 73% were men (*n* = 280). Penetrating injuries accounted for 3.4% (*n* = 13). The median injury severity score of the patients who required transfusion (subgroups PHBT + /MT + and PHBT − /MT +) was 33 (IQR 25–75: 22–45) and for the patients who did not require transfusion (subgroups PHBT + /MT − and PHBT − /MT −) it was 14 (IQR 25–75: 9–23), p ˂ 0,001 (the criterion is the activation of MT after admission to the ED).

**Fig. 3:**
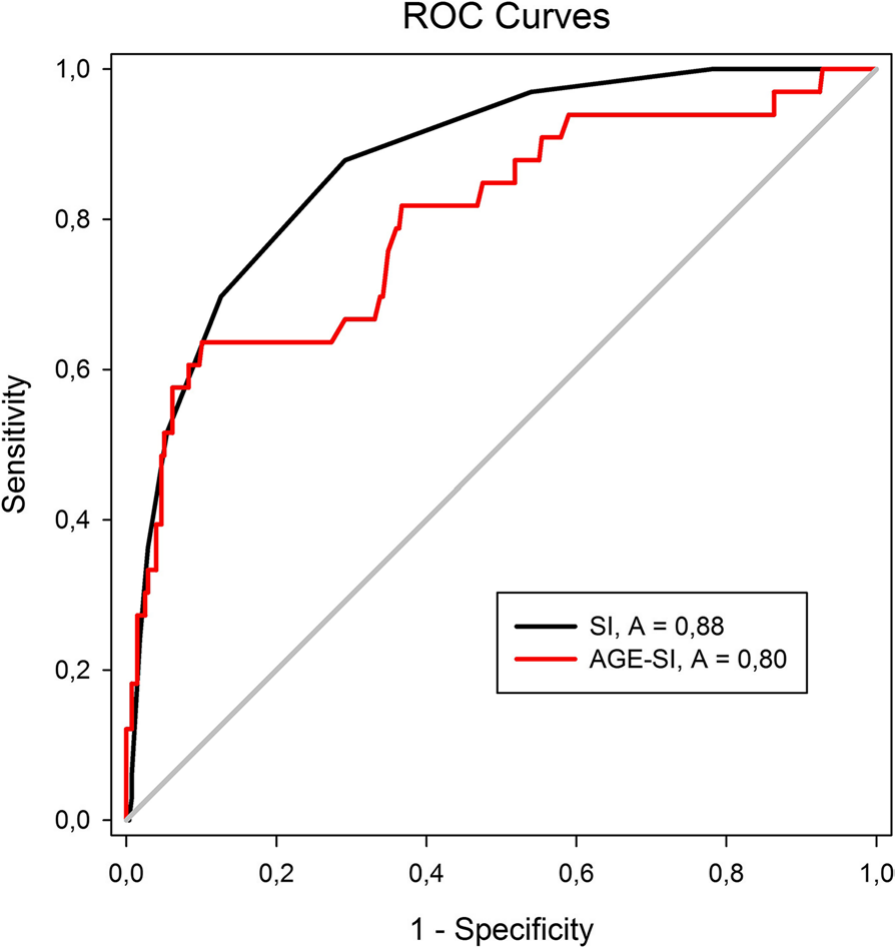
Receiver Operating Characteristic Curves for SI and Age-SI

**Fig. 4:**
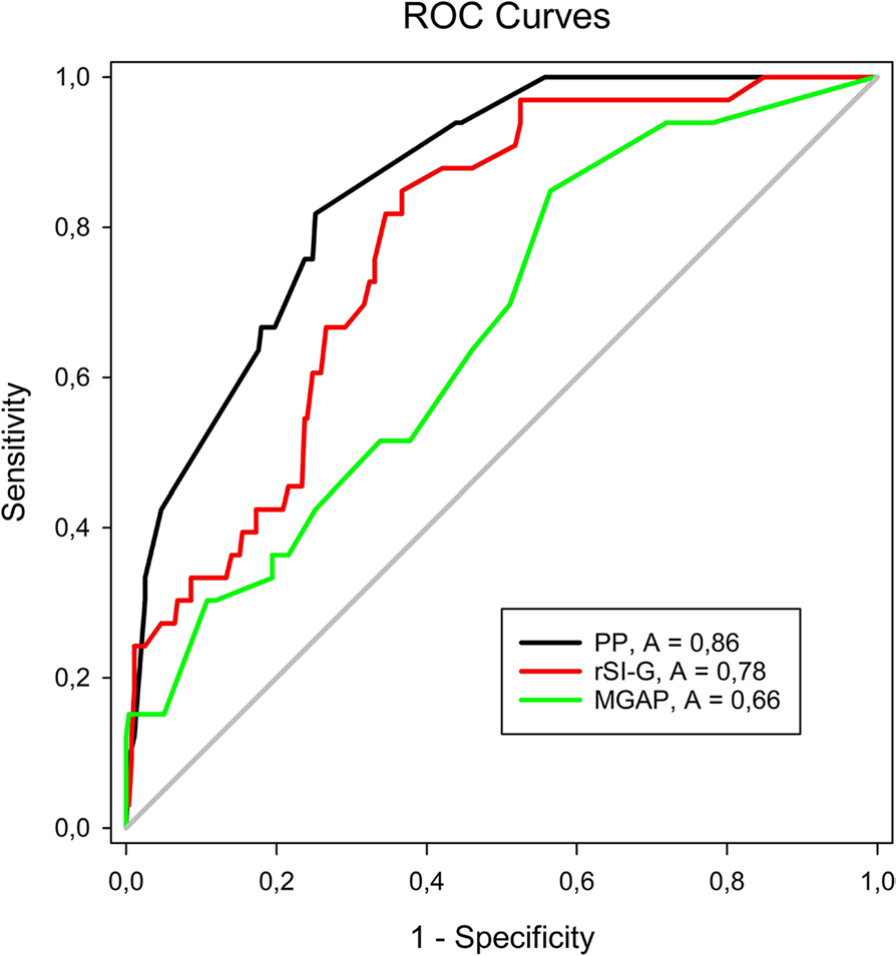
Receiver Operating Characteristic Curves for PP, rSI-G and MGAP

The median age was 44 years (IQR 25–75: 24–60). The median interval from the dispatch center’s first call to the “ED door” was 59 min (IQR 25–75: 42–76), and the median transport time to the trauma center was 15 min (IQR 25–75: 12–19 min). No haemolytic reactions were observed after transfusion administrations during the study. The highest area under curve (AUC; > 0.8) value was found for the SI scoring system (AUC 0.88; 95% CI 0.82–0.93). The remaining scoring system with an AUC > 0,8 was the PP (AUC 0.85 with 95% CI 0.79–0.91). On the contrary, the lowest validity for the identification of patients with the PHBT requirement was discovered in the MGAP scoring system (AUC 0.66; 95% CI 0.57–0.76), respectively, rSI–G (AUC 0.77; 95% CI 0.70–0.85) (Table 2, Figs. 3, 4).

**Table 2:** Comparison of the predictive value and optimal cut-off

Scoring system	AUC	95% CI	SE	Cut-off	Sensitivity (%)	Specificity (%)
SI	0.88	0.82–0.93	0.03	0.85	70	87
PP	0.85	0.79–0.91	0.03	40	76	76
AGE–SI	0.79	0.71–0.89	0.05	35	67	69
rSI–G	0.77	0.70–0.85	0.04	16	73	67
MGAP	0.66	0.57–0.76	0.05	24.5	64	54

## Discussion

This retrospective analysis of traumatized patients’ data available in the HEMS and the University Hospital registers revealed that the shock index (AUC 0.88), reversed shock index (AUC 0.88) and pulse pressure (AUC 0.86) are suitable scoring systems for the identification of high-risk patients requiring PHBT.

The concept of pre-hospital transfusion administration is based on experience from the Vietnam war and has been optimized during further conflicts. The decrease in long-term mortality has been confirmed compared to patients who did not receive PHBT or received a late transfusion [17]. PHBT has also been introduced into some European Emergency Medical Services (EMS) procedures, respectively, the HEMS. The Czech Republic (resp. the HEMS of the Hradec Kralove Region) is one of eleven EMS/HEMS in European countries where this practice has been applied [18]. The pre-hospital teams face the challenge of identifying patients for PHBT. The identification criteria between countries differ. Based on the survey conducted among European countries, the main identifier for PHBT is major trauma, shock, and prolonged entrapment in unstable patients [18]. According to the review, which involved 22 PHBT studies by Shand et al., the physiological criterion most frequently assessed is systolic blood pressure (SBP) (varied between < 70 and < 90 mmHg), tachycardia (varied between > 108 and > 130/min) or no radial pulse. The mechanism of injury (penetrating injury or amputation above the knee/elbow) was included in 5 studies as an indication criterion. In 4 studies, the criteria for PHBT were not identified and in six studies, the criteria for PHBT were not quantified [19]. Rijnhout et al. in the review of 32 studies also recorded the lactate test (> 5), haemoglobin value (< 7 g/dl), the estimate of blood loss (> 500 ml), capillary return (> 2 s) and clinical gestalt as indication criteria [6].

The link to massive transfusion activation in the in-hospital phase has been proved in the scoring systems. Many of them, except physiological function values, use the results of lab examinations, imaging, or time-consuming numerical processes that are difficult to use in the pre-hospital setting, especially in EMS/HEMS that do not use these complements [13, 14]. Colleagues from Spain conducted a retrospective analysis in 2019 on the topic of prehospital prediction of massive bleeding using scoring systems. The best results were achieved by a score that had at least 6 variables, including BE, serum Hb, or FAST performed during transport to the ED: Emergency Transfusion Score (ETS; AUC 0.85), Trauma Associated Severe Haemorrhage (TASH) and the Prince of Wales Hospital score (AUC 0.82) [20]. Since many pre-hospital systems do not use imaging and laboratory complement, we focused on scoring systems using readily available vital signs values.

The shock index, defined as heart rate/SBP, is a better predictor of trauma outcome than vital signs alone [10, 11]. SI can be used, according to the European Guideline on the management of major bleeding and coagulopathy after trauma, to assess the seriousness of hypovolemic shock [21]. Vandrome and colleagues have proved the increasing risk of MT requirement if the SI value rises over 0.9 even in relatively normotensioned patients [22]. El-Menyar et al. defined the optimal cut-off point as 0.81 for predicting MT in trauma patients (sensitivity 85%, specificity 64%) [23]. Some clinicians prefer the characteristic of unstable hemodynamic status as a lower SBP than HR and not a higher HR than SBP. Kimura et al. considered reversed shock index (rSI – ratio SBP to HR) and GCS together (rSI–G: rSI multiplied value of GCS) and proved that rSI–G was a better predictor of in-hospital mortality and 24-h blood transfusion than SI [24]. Another research group from South Korea found that rSI–G is a strong predictor of massive transfusion initiation in the ED with median rSI–G 6.47 (IQR 25–75: 3.80–12.24) [25]. The next derivative of SI, the age-related shock index (AGE–SI), was introduced to improve the accuracy of SI. Rau et al. found the AGE–SI cut-off point 36.95 to predict the requirement for MT (AUC 0.627) [26]. Pulse pressure (PP) is defined as the difference between diastolic and systolic blood pressure. The value of PP narrows in bleeding patients as a response to decreased intravascular volume. The main purpose of the research led by Priestley et al. was to determine whether a narrowed PP in a normotensive patient (SBP ≥ 90 mmHg) is an independent predictor of bleeding. They found that the mean PP was significantly lower in the group with acute haemorrhage (AH) compared to the group without AH (39 ± 18 mmHg vs 53 ± 19 mmHg, p ˂ 0.0001). The analysis identified a significantly higher risk of AH at the PP cut-off of 55 mmHg (*p* = 0.005 AUC 0.955) in patients 61 years or older vs 40 mmHg (*p* < 0.0001, AUC 0.940) in patients from 16 to 60 years [27]. The median age value in our analysis was 44 (IQR 25–75: 24–60) and thus we consider the PP cut-off of 40 mmHg as referenced (sensitivity 75%, specificity 76%). The MGAP system (mechanism of injury, GCS, age, and systolic blood pressure) was introduced in France in 2010 for physician-staffed EMS crews and was originally defined for the prediction of in-hospital mortality. Based on the acquired values, three risk groups were defined: low (23–29 points), intermediate (18–22 points) and high risk (< 18 points). In the derivation cohort, the mortality was 2.8%, 15% and 48%, respectively [28]. The HEMS crew in the Czech Republic is staffed with a physician, therefore, this system was included in our analysis in order to verify its applicability in the pre-hospital phase. In the prediction of the patient's PHBT requirement, there was the lowest AUC value (AUC 0.66; 95% CI 0.57–0,76; cut-off 24.5) and the suitability of MGAP for PHBT was not demonstrated.

To the best of our knowledge, the comparison of the above-mentioned scoring systems has not been carried out in association with the initiation of PHBT.

The parameters in the pre-hospital phase show dynamics and variability, e.g., in connection to age and make the final decision difficult. Thus, whether a unified algorithm for PHBT is possible to develop arises. Nevertheless, the use of easily calculated SI and PP scoring systems allow the range of available criteria for the PHBT to be extended, the decision-making process to be optimized and minimize the risk of unnecessary administration (of an expensive and rare commodity) or, on the contrary, the miscalculation of patients who can profit from PHBT administration.

### Limitations

Our study has several limitations. Firstly, it was a single-center study, thus these results are difficult to generalize in clinical practice. Secondly, it was a retrospective study with some patients missing data. The evaluated period was 30 months and involved a relatively small cohort of patients to whom PHBT was administered. PHBT was established at our workplace in the middle of 2018 and is considered a fairly new method, and the results presented reveal our first experience. Thirdly, we did not carry out stratification of patients according to blunt or penetrating injuries. We also did not differentiate the subgroups of patients with head trauma. These patients with serious head trauma and clinical manifestation of an increase in SBP and a decrease in heart rate could have an impact on score calculation. We did not perform further analysis in relation to emergency surgery or angioembolization, ICU length of stay or mortality. Given that the main objective was to identify patients who required PHBT prior to a hospital-administrated massive transfusion, we did not perform a detailed analysis of patients from the PHBT + /MT − subgroup (8 patients). Some of these patients may have benefited from one or two units of blood products administered in the pre-hospital phase and then no longer required transfusions after reaching the hospital. This may have limited our ability to identify predictors for patients who may benefit from just a prehospital blood transfusion. At the same time, we also did not consider the age of the patients, when a higher threshold for the physiological value of SBP can be assumed in higher age categories. We also did not consider comorbidities with associated medications (mainly antiarrhythmics) that could influence the predictive value of the indexes.

## Conclusion

Shock index and pulse pressure are suitable scoring systems for the prediction of patients with PHBT requirements. Further research is required to determine the optimal threshold values of the scoring systems and their usefulness in predicting pre-hospital life-threatening haemorrhages.

## Data Availability

De-identified datasets used during the current study are available from the corresponding author upon reasonable request.

## Abbreviations

AGE–SI: Age multiplied by shock index
DBP: Diastolic blood pressure
ED: Emergency department
EMS: Emergency medical service
ETS: Emergency transfusion score
GCS: Glasgow Coma Scale
HEMS: Helicopter emergency medical service
HR: Heart rate
LTOWB: Low titer group O whole blood
MGAP: Mechanism of injury, GCS, age, systolic blood pressure
MT: Massive transfusion
PHBT: Pre-hospital blood transfusion
PP: Pulse pressure
PRBC: Packed red blood cells
rSI–G: Reverse shock index multiplied by GCS
SBP: Systolic blood pressure
SI: Shock index
TASH: Trauma associated severe haemorrhage
